# Potential of cell-free hemoglobin and haptoglobin as prognostic markers in patients with ARDS and treatment with veno-venous ECMO

**DOI:** 10.1186/s40560-023-00664-5

**Published:** 2023-04-20

**Authors:** Victoria Bünger, Oliver Hunsicker, Alexander Krannich, Felix Balzer, Claudia D. Spies, Wolfgang M. Kuebler, Steffen Weber-Carstens, Mario Menk, Jan A. Graw

**Affiliations:** 1 Department of Anesthesiology and Intensive Care Medicine CCM / CVK, Charité – Universitätsmedizin Berlin, corporate member of Freie Universität Berlin and Humboldt-Universität zu Berlin, Augustenburger Platz 1, 13353 Berlin, Germany; 2 ARDS/ECMO Centrum Charité, Charité – Universitätsmedizin Berlin, Berlin, Germany; 3 Clinical Trial Office, Charité – Universitätsmedizin Berlin, Berlin, Germany; 4 Department Analytics, TCC GmbH, Hamburg, Germany; 5 Institute of Medical Informatics, Charité – Universitätsmedizin Berlin, Berlin, Germany; 6 Institute of Physiology, Charité – Universitätsmedizin Berlin, Berlin, Germany; 7 Department of Anesthesiology and Intensive Care Medicine, University Hospital “Carl Gustav Carus”, Technische Universität Dresden, Dresden, Germany; 8 Department of Anesthesiology and Intensive Care Medicine, Universitätsklinikum Ulm, Ulm University, Ulm, Germany

**Keywords:** Lung injury, Organ replacement technology, Hemolysis, Erythrocyte pathology, Red cell pathology, Hemoglobin scavenger

## Abstract

**Background:**

Hemolysis is associated with increased mortality in patients with sepsis, ARDS, or therapy with extracorporeal membrane oxygenation (ECMO). To quantify a critical threshold of hemolysis in patients with ARDS and treatment with veno-venous ECMO, we aimed to identify cutoff values for cell-free hemoglobin (CFH) and haptoglobin (Hp) plasma concentrations associated with a significant increase in ICU mortality.

**Methods:**

Patients with ARDS admitted to a tertiary ARDS referral center between 01/2007 and 12/2018 and treatment with veno-venous ECMO were included. Cutoff values for mean CFH (mCFH) and mean Hp (mHp) plasma concentrations dividing the cohort into groups with significantly different ICU mortalities were calculated and patient characteristics were compared. A multiple logistic regression model with stepwise backward variable selection was included. In addition, cutoff values for vulnerable relative timespans for the respective CFH and Hp concentrations were calculated.

**Results:**

A quantitative cutoff value of 11 mg/dl for mCFH separated the cohort (*n* = 442) regarding ICU mortality (mCFH ≤ 11 mg/dl: 38%, [95%-CI: 32.22–43.93] (*n* = 277) vs. mCFH > 11 mg/dl: 70%, [61.99–76.47] (*n* = 165), *p* < 0.001). Analogously, a mHp cutoff value ≤ 0.39 g/l was associated with a significant increase in ICU mortality (mHp ≤ 0.39 g/l: 68.7%, [60.91–75.61] (*n* = 163) vs. mHp > 0.39 g/l: 38.7%, [33.01–44.72] (*n* = 279), *p* < 0.001). The independent association of ICU mortality with CFH and Hp cutoff values was confirmed by logistic regression adjusting for confounders (CFH Grouping: OR 3.77, [2.51–5.72], *p* < 0.001; Hp Grouping: OR 0.29, [0.19–0.43], *p* < 0.001). A significant increase in ICU mortality was observed when CFH plasma concentration exceeded the limit of 11 mg/dl on 13.3% of therapy days (≤ 13.3% of days with CFH > 11 mg/dl: 33%; [26.81–40.54] (*n* = 192) vs. > 13.3% of days with CFH > 11 mg/dl: 62%; [56.05–68.36] (*n* = 250), *p* < 0.001). Analogously, a mortality increase was detected when Hp plasma concentration remained ≤ 0.39 g/l for > 18.2% of therapy days (≤ 18.2% days with Hp ≤ 0.39 g/l: 27%; [19.80–35.14] (*n* = 138) vs. > 18.2% days with Hp ≤ 0.39 g/l: 60%; [54.43–65.70] (*n* = 304), *p* < 0.001).

**Conclusions:**

Moderate hemolysis with mCFH-levels as low as 11 mg/dl impacts mortality in patients with ARDS and therapy with veno-venous ECMO. Furthermore, a cumulative dose effect should be considered indicated by the relative therapy days with CFH-concentrations > 11 mg/dl. In addition, also Hp plasma concentrations need consideration when the injurious effect of elevated CFH is evaluated.

**Supplementary Information:**

The online version contains supplementary material available at 10.1186/s40560-023-00664-5.

## Background

Hemolysis is a common complication in intensive care patients [1–4]. Among various contributing factors, significant associations with patient-specific disease conditions, such as septic shock or the acute respiratory distress syndrome (ARDS), as well as therapy with extracorporeal life support systems, hemodialysis, or the transfusion of packed red blood cells (PRBCs) that have been stored for prolonged intervals, have been observed [2, 5–9]. Intravascular hemolysis occurs when erythrocytes are damaged by immunological or mechanical effects. The intracellular contents of the red cells are released into the patient´s vasculature leading to various adverse reactions including oxidation, inflammation and platelet aggregation [10–12]. Intravascular liberated cell-free hemoglobin (CFH) can scavenge nitric oxide (NO) in the endothelium subsequently causing vasoconstriction and reduction of blood flow [13, 14]. In addition, reactive oxygen species are generated through Haber–Weiss- and Fenton-reactions mediated by hemoglobin, heme and iron [15, 16]. CFH can also cause direct cytotoxic injury to cell membranes, plasma proteins and lipids [11, 17–19]. Furthermore, elevated plasma concentrations of CFH have been observed to be associated with direct organ injuries, such as renal failure, intestinal mucosal damage, or lung injury [2, 4, 20, 21]. Endogenous protective systems counteracting CFH include the hepatic derived plasma proteins haptoglobin (Hp) and hemopexin, which can bind to CFH and free heme, respectively [22, 23]. Besides CFH itself, Hp and LDH are sensitive diagnostic markers for hemolysis that can be measured routinely to monitor red cell break down [4, 24–27].

An association between elevated CFH levels and mortality has been observed in patients with sepsis, sickle cell disease, or after cardiac surgery with cardiopulmonary bypass [2, 26, 28]. Patients with a severe ARDS and treatment with veno-venous extracorporeal membrane oxygenation (V-V ECMO) have various risk factors that might contribute to hemolysis [5]. In addition, these patients appear particularly susceptible to CFH and its degradation products that might contribute to further lung injury [29, 30]. Previous studies on patients with ARDS receiving treatment with ECMO have also demonstrated an association between elevated CFH plasma concentrations and mortality [7, 31–33]. However, these studies consisted of small patient cohorts, considered only CFH values at 24 h after ECMO initiation or the maximum CFH value during the course of treatment [7, 31–33]. Therefore, only limited conclusions can be drawn concerning the impact of hemolysis during ICU therapy.

For patients with ARDS receiving treatment with ECMO, an association of renal failure with elevated CFH levels at ECMO initiation was observed [4]. Furthermore, hemolysis-associated renal failure in this patient cohort was also inversely associated with Hp plasma concentrations [4]. These data not only suggest a protective effect of Hp when increased CFH plasma levels are present but also indicate that Hp concentrations should be taken into consideration when effects of hemolysis are evaluated.

This study aims to reach a better understanding of the extent of hemolysis required to increase mortality in patients with ARDS and V-V ECMO. Cutoff values of mean CFH and Hp plasma concentrations associated with a significant increase in ICU mortality were identified. Apart from the mean concentrations of CFH and Hp over the course of ICU therapy, limits for critical timespans of relevant hemolysis could be determined. Notably, timespans of critically elevated CFH might be considerably shorter than the total therapy time yet may nonetheless increase mortality due to cumulative dose effects. To evaluate the effect of short phases of hemolysis, maximum CFH and minimum Hp cutoff values were calculated.

## Methods

### Study design and setting

**Fig. 1:**
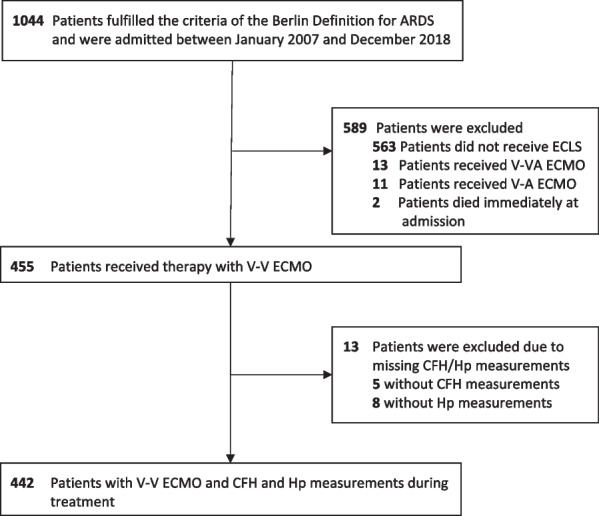
Patient flow diagram. ECLS—extracorporeal life support; ECMO—extracorporeal membrane oxygenation with veno-veno-arterial (V-VA), venoarterial (V-A), or venovenous (V-V) cannulation

This retrospective cohort study includes adult ARDS patients receiving treatment with V-V ECMO at the tertiary ARDS center of the Department of Anesthesiology and Intensive Care Medicine, Charité – Universitätsmedizin Berlin, who were admitted between January 2007 and December 2018. All patients with routinely monitored CFH and haptoglobin plasma concentrations were included in the study (Fig. 1). Patients were excluded from analysis if they received treatment with V-VA or V-A ECMO or if they died within the first 6 h after admission. The study was approved by the Medical Ethics Committee of the Charité – Universitätsmedizin Berlin (No. EA2/019/19).

### Data sources

All data required for this study were extracted from the two electronic patient management systems used at the hospital (SAP, Walldorf, Germany and COPRA, Sabachswalden, Germany) [34]. Daily measurements of CFH and Hp plasma concentrations in ARDS patients receiving therapy with V-V ECMO occurred as described previously [4].

### Endpoints

The primary objective was to identify cutoff values of mean CFH and mean Hp plasma concentrations that define a significant increase in ICU mortality in patients with ARDS and treatment with V-V ECMO. The secondary objective was to identify cutoff values for intervals of elevated CFH and lowered Hp levels associated with significant differences in ICU mortality.

### Statistical analysis

Mean CFH and mean Hp plasma concentrations during therapy with V-V ECMO were obtained as parameters to detect hemolysis over the course of treatment. Mean CFH and mean Hp values were defined as the arithmetic mean of all daily measured CFH or Hp values of each individual patient during therapy with V-V ECMO. Cutoff values were calculated with recursive binary partitioning using conditional inference trees and splitting the cohort into two groups with the most significantly different mortality [35]. Patients were grouped according to the calculated CFH and Hp cutoff values. Statistical testing for differences in baseline characteristics was performed with Students’ *T* test for normally distributed continuous data and with exact Mann–Whitney *U* test for non-normally distributed continuous data. For frequencies, Fisher’s exact test was used. A two-tailed *p* value of 0.05 was considered statistically significant. To adjust for all statistically significantly different baseline characteristics and ventilation parameters between the groups, a multiple logistic regression model using backward variable selection based on the Akaike information criterion (AIC) was chosen. For visualization of predicted ICU mortality, a multiple logistic regression model including the mean CFH and mean Hp as response variables was utilized. To detect a cutoff value for a critical time span subjected to an elevated CFH, we defined the CFH time component as the percentage of days spent over the afore calculated critical CFH value in relation to all therapy days. Accordingly, the Hp time component was defined as the percentage of days the Hp value was under the calculated cutoff value in relation to all therapy days. For missing CFH (13.5%) or Hp (22.5%) values, mean person substitution was chosen as single imputation method, and were substituted with the patient specific mean CFH or mean Hp value, respectively. [36] All analyses were performed with R software, version 4.2.0 (R Project for Statistical Computing, Vienna, Austria).

## Results

A total of 455 ARDS patients admitted to the tertiary ARDS referral center between January 2007 and December 2018 received treatment with V-V ECMO (Fig. 1). All patients with measured CFH and Hp plasma concentrations were included in the analysis and mean CFH and mean Hp plasma levels during therapy with V-V ECMO were calculated (Fig. 1).

**Fig. 2:**
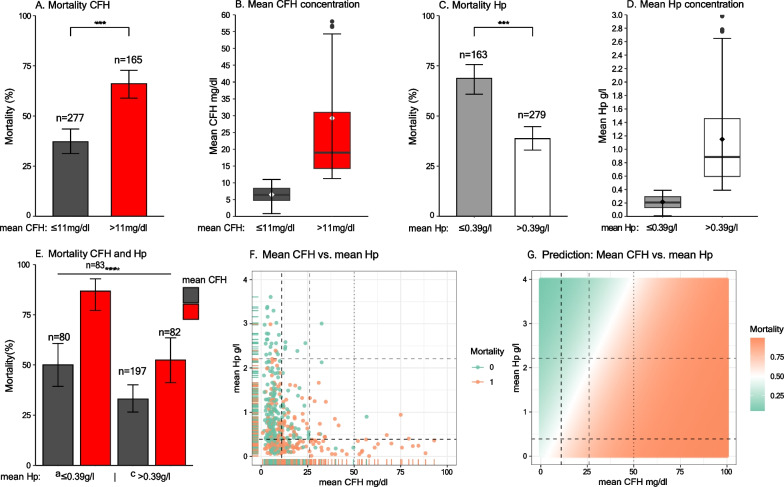
**A** Mortality by CFH group. **B** Mean CFH concentration according to CFH group. **C** Mortality by Hp group. **D** Mean Hp concentration according to Hp group. **E** Mortality by CFH and Hp cutoff. **F** Scatter plot of the distribution of patients according to their mean CFH and mean Hp values, colored by ICU mortality. The calculated mean CFH limits (11 mg/dl and 26 mg/dl) and mean Hp limit (0.39 g/l and 2.21 g/l) are given as dashed lines. The commonly used CFH limit of 50 mg/dl is shown as dotted line. The dark dashed lines representing the most significant cutoff values of 11 mg/dl for CFH and 0.39 g/l for Hp discriminate the patients into the four groups shown in **E**. **G** Prediction of ICU mortality based on mean CFH and mean Hp values. Dashed lines represent the calculated CFH (11 mg/dl and 26 mg/dl) and Hp (0.39 g/l and 2.21 g/l) limits, the dotted line indicates the CFH value of 50 mg/dl

Through recursive binary partitioning, a cutoff value for CFH of 10.714 mg/dl, rounded to 11 mg/dl, was found to divide the population into two groups with the most significant difference in ICU mortality (mean CFH ≤ 11 mg/dl: 38%, [95% CI, 32.22–43.93], *n* = 277 vs. mean CFH > 11 mg/dl: 70%, [61.99–76.47], *n* = 165, *p* < 0.001, Fig. 2A). The mean CFH of the resulting groups discriminated significantly (mean CFH ≤ 11 mg/dl: median 6.42 mg/dl [IQR, 4.75–8.35] vs. mean CFH > 11 mg/dl: 19.00 mg/dl [14.29–31.00], *p* < 0.001), as illustrated in Fig. 2B. A second cutoff value of 25.571 mg/dl, rounded to 26 mg/dl, was found to further divide the second group (mean CFH 11–26 mg/dl: 62%, [95% CI, 51.91–70.50], *n* = 112 vs. mean CFH > 26 mg/dl: 87%, [74.05–94.09], *n* = 53, *p* = 0.002). For further analysis, only the lower CFH cutoff value was taken into consideration. A simple logistic regression showed a significant association of the grouping with ICU-mortality (OR 3.77; [95% CI, 2.51–5.72] *p* < 0.001).

With a complementary approach, Hp plasma concentrations were analyzed. A Hp plasma level of 0.388 g/l, rounded to 0.39 g/l, was identified as cutoff value dividing the population into two groups with a significantly different ICU-mortality (mean Hp ≤ 0.39 g/l: 68.7%, [95% CI, 60.91–75.61], *n* = 163 vs. mean Hp > 0.39 g/l: 38.7%, [33.01–44.72], *n* = 279, *p* < 0.001, Fig. 2C). A second cutoff value of 2.205 g/l, rounded to 2.21 g/l, further divided the second group (mean Hp 0.39–2.21 g/l: 42.0%, [95% CI, 35.86–48.39], *n* = 250 vs. mean Hp > 2.21 g/l: 10.3%, [2.71–28.50], *n* = 29, *p* = 0.001). The Hp groups resulting from the first and most significant cutoff value discriminated significantly in their mean Hp concentration (mean Hp ≤ 0.39 g/l: median 0.21 g/l [IQR, 0.13–0.29] vs. mean Hp > 0.39 g/l: median 0.89 g/l [0.60–1.46], *p* < 0.001, Fig. 2D). The simple logistic regression showed a significant inverse association of the grouping with ICU-mortality (OR 0.29; [95% CI, 0.19–0.43], *p* < 0.001).

Figure 2E illustrates mean CFH-dependent mortality differences separated by the previously detected Hp thresholds (mortality for mean Hp ≤ 0.39 g/l and CFH ≤ 11 mg/dl: 50.0%, [95% CI, 39.30–60.70, *n* = 80, for mean Hp ≤ 0.39 g/l and CFH > 11 mg/dl: 86.7%, [77.11–92.88], *n* = 83, for mean Hp > 0.39 g/l and CFH ≤ 11 mg/dl: 33.0%, [26.57–40.09], *n* = 197, and for mean Hp > 0.39 g/l and CFH > 11 mg/dl: 52.4%, [41.18–63.47], *n* = 82, *p* < 0.001). As suggested by the previous analysis and illustrations, mean CFH and Hp levels were inversely and independently associated with ICU mortality (Fig. 2F). Prediction of ICU mortality based on a multiple logistic regression model considering the mean CFH and mean Hp values is illustrated in Fig. 2G.

A comparison of baseline characteristics between the two CFH groups revealed significant differences for SOFA, APACHE, SAPS, and RASS scores at ARDS onset, various ventilation parameters at ECMO initiation, the incidence of septic shock, renal replacement therapy (RRT), number of transfused PRBCs, lactate levels, and therapy with inhaled nitric oxide (Table 1). Analogously, the two groups discriminated by the first Hp cutoff value differed significantly in baseline characteristics BMI, CCI, SOFA, SAPS, and APACHE scores at ARDS onset as well as ARDS etiology, pH, and pulmonary compliance at ECMO initiation, incidence of septic shock, RRT, number of transfused PRBCs, lactate levels, and therapy with inhaled nitric oxide (Table 2).

**Table 1:** Baseline characteristics according to CFH grouping

Characteristic	Mean CFH < 11 mg/dl (*n* = 277)	Mean CFH > 11 mg/dl (*n* = 165)	*P* value
Mean CFH (mg/dl)	6.42 [4.75, 8.35]	19.00 [14.29, 31.00]	< 0.001
ICU mortality, *n* (%)	105 (37.9)	115 (69.7)	< 0.001
ECMO duration (days)	14.00 [8.00, 27.00]	13.00 [6.00, 26.00]	0.118
Age (years)	49.00 [38.00, 61.00]	48.00 [36.00, 59.00]	0.386
Male sex, *n* (%)	180 (65.0)	104 (63.0)	0.683
Body mass index (kg/m^2^)	24.84 [21.60, 29.39]	25.95 [22.86, 30.86]	0.168
PBW (kg)	70.46 [59.63, 74.99]	67.75 [59.63, 74.99]	0.289
Charlson comorbity index	2.00 [1.00, 4.00]	2.00 [1.00, 5.00]	0.926
Immunocompromised, *n* (%)	68 (24.5)	48 (29.1)	0.315
SOFA at ARDS onset	11.00 [8.00, 14.00]	14.00 [11.00, 16.00]	< 0.001
APACHE at ARDS onset	26.00 [20.00, 33.00]	29.00 [23.00, 38.00]	0.001
SAPS at ARDS onset	53.00 [38.00, 68.00]	61.00 [46.00, 76.00]	< 0.001
RASS at ARDS onset	− 5.00 [− 5.00, − 4.00]	− 5.00 [− 5.00, − 4.50]	0.001
Pulmonary origin, *n* (%)	253 (91.3)	142 (86.1)	0.110
ARDS severity, *n* (%)			0.924
Mild	1 (0.4)	1 (0.6)	
Moderate	18 (6.5)	9 (5.5)	
Severe	258 (93.1)	155 (93.9)	
ARDS etiology, *n* (%)			0.058
Pneumonia	195 (70.4)	110 (66.7)	
Aspiration	18 (6.5)	22 (13.3)	
Other	64 (23.1)	33 (20.0)	
ECMO initiation (ICU day)	0.00 [0.00, 1.00]	0.00 [0.00, 0.00]	0.416
Ventilation parameters at ECMO Initiation			
PaO_2_/FiO_2_ (mmHg)	68.12 [54.72, 91.32]	63.65 [50.68, 76.53]	0.054
PaO_2_ (mmHg)	66.50 [54.45, 86.85]	61.95 [49.98, 73.70]	0.049
PaCO_2_ (mmHg)	66.70 [55.10, 84.90]	64.05 [51.98, 79.15]	0.314
pH	7.25 [7.17, 7.35]	7.22 [7.13, 7.29]	0.009
PIP (cmH_2_O)	38.00 [34.15, 43.00]	41.31 [37.30, 46.27]	< 0.001
Pplat (cmH_2_O)	36.00 [31.00, 38.93]	37.33 [34.89, 41.00]	0.001
PEEP (cmH_2_O)	18.00 [14.00, 20.00]	20.00 [16.30, 22.00]	< 0.001
Driving pressure (cmH_2_O)	17.60 [13.05, 20.70]	17.97 [15.27, 22.13]	0.074
Respiratory rate (breaths/min)	25.00 [21.04, 27.94]	23.55 [20.00, 27.00]	0.295
Compliance (ml/cmH_2_O)	29.76 [19.86, 44.55]	29.36 [21.64, 45.27]	0.847
Tidal volume (ml)	368.09 [264.67, 473.33]	392.69 [296.90, 528.57]	0.129
Septic shock, *n* (%)	151 (54.5)	127 (77.0)	< 0.001
RRT, *n* (%)	160 (57.8)	132 (80.0)	< 0.001
PRBC units transfused (number)	17.00 [8.00, 31.00]	23.00 [13.00, 42.00]	< 0.001
Lactate (mg/dl)	16.00 [10.00, 35.00]	27.00 [14.00, 87.00]	< 0.001
Further rescue therapies, *n* (%)			
Inhaled nitric oxide	192 (69.3)	136 (82.4)	0.002
Prone positioning	203 (73.3)	127 (77.0)	0.429

*CFH* cell-free hemoglobin, *SOFA* sequential organ failure assessment, *APACHE* acute physiology and chronic health evaluation, *SAPS* simplified acute physiology score, *RASS* Richmond Agitation–Sedation Scale, *PIP* peak inspiratory pressure, *Pplat* plateau pressure, *PEEP* positive end-expiratory pressure, *RRT* renal replacement therapy, *PRBC* packed red blood cells. Data are expressed as mean (SD), median (25%, 75% quartiles) or frequencies (%), as appropriate

**Table 2:** Baseline characteristics according to Hp grouping

Characteristic	Mean Hp 0.39 g/l (*n* = 163)	Mean Hp 0.39 g/l (*n* = 279)	*P* value
Mean Hp (g/l)	0.21 [0.13, 0.29]	0.89 [0.60, 1.46]	< 0.001
ICU mortality, *n* (%)	112 (68.7)	108 (38.7)	< 0.001
ECMO duration (days)	15.00 [5.50, 26.50]	13.00 [7.00, 26.50]	0.601
Age (years)	50.00 [34.00, 60.00]	48.00 [38.00, 61.00]	0.740
Male sex, *n* (%)	103 (63.2)	181 (64.9)	0.758
Body mass index (kg/m^2^)	24.69 [21.03, 28.63]	25.77 [22.17, 30.86]	0.045
PBW (kg)	67.75 [58.95, 74.99]	70.46 [59.63, 74.99]	0.233
Charlson comorbity index	3.00 [1.00, 5.00]	2.00 [0.00, 4.00]	0.022
Immunocompromised, *n* (%)	47 (28.8)	69 (24.7)	0.371
SOFA at ARDS onset	13.00 [10.00, 16.00]	11.00 [8.50, 14.00]	0.004
APACHE at ARDS onset	29.00 [23.50, 36.00]	27.00 [20.00, 33.00]	0.010
SAPS at ARDS onset	61.00 [41.00, 75.50]	55.00 [40.00, 68.00]	0.016
RASS at ARDS onset	-5.00 [-5.00, -4.00]	-5.00 [-5.00, -4.00]	0.725
Pulmonary origin, *n* (%)	141 (86.5)	254 (91.0)	0.151
ARDS severity, *n* (%)			0.199
Mild	2 (1.2)	0 (0.0)	
Moderate	11 (6.7)	16 (5.7)	
Severe	150 (92.0)	263 (94.3)	
ARDS etiology, *n* (%)			0.001
Pneumonia	96 (58.9)	209 (74.9)	
Aspiration	24 (14.7)	16 (5.7)	
Other	43 (26.4)	54 (19.4)	
ECMO initiation (ICU day)	0.00 [0.00, 1.00]	0.00 [0.00, 0.00]	0.056
Ventilation parameters at ECMO Initiation			
PaO_2_/FiO_2_ (mmHg)	64.50 [51.62, 85.37]	68.50 [54.80, 86.26]	0.221
PaO_2_ (mmHg)	63.05 [50.75, 80.80]	66.10 [53.80, 81.00]	0.206
PaCO_2_ (mmHg)	68.90 [55.30, 83.18]	62.60 [53.50, 82.20]	0.393
pH	7.22 [7.12, 7.29]	7.25 [7.17, 7.34]	0.012
PIP (cmH_2_O)	40.20 [36.00, 44.27]	39.00 [34.93, 43.00]	0.100
Pplat (cmH_2_O)	36.07 [33.07, 40.50]	36.00 [32.00, 39.56]	0.440
PEEP (cmH_2_O)	18.40 [14.82, 21.00]	19.00 [15.00, 21.00]	0.460
Driving pressure (cmH_2_O)	18.27 [14.65, 21.42]	17.80 [14.00, 21.00]	0.398
Respiratory rate (breaths/min)	24.00 [21.00, 27.20]	25.00 [20.49, 27.67]	0.966
Compliance (ml/cmH_2_O)	27.20 [19.00, 40.00]	32.83 [21.84, 47.91]	0.027
Tidal volume (ml)	349.73 [241.14, 499.75]	383.30 [297.38, 504.91]	0.144
Septic shock, *n* (%)	120 (73.6)	158 (56.6)	< 0.001
RRT, *n* (%)	124 (76.1)	168 (60.2)	0.001
PRBC units transfused (number)	26.00 [15.50, 43.50]	16.00 [8.00, 31.00]	< 0.001
Lactate (mg/dl)	26.00 [11.00, 78.00]	17.00 [11.00, 35.00]	< 0.001
Further rescue therapies, *n* (%)			
Inhaled nitric oxide	131 (80.4)	197 (70.6)	0.025
Prone positioning	121 (74.2)	209 (74.9)	0.910

*Hp* haptoglobin, *SOFA* sequential organ failure assessment, *APACHE* acute physiology and chronic health evaluation, *SAPS* simplified acute physiology score, *RASS* Richmond Agitation–Sedation Scale, *PIP* peak inspiratory pressure, *Pplat* plateau pressure, *PEEP* positive end-expiratory pressure, *RRT* renal replacement therapy, *PRBC* packed red blood cells. Data are expressed as mean (SD), median (25%, 75% quartiles) or frequencies (%), as appropriate

To adjust for all potential confounders, all significantly different variables from the CFH- and Hp-discriminated groups were included in a backward stepwise variable selection. The resulting multivariable logistic regression model based on the lowest AIC included the four variables CFH grouping, Hp grouping, BMI, and pH at ECMO initiation (Table 3). Herein, significant association of the calculated CFH (CFH Grouping: OR 3.77, [95% CI, 2.51–5.72], *p* < 0.001, adj. OR 2.17, [95% CI, 1.11–4.32], *p* = 0.025) and Hp cutoff values (Hp Grouping: OR 0.29, [0.19–0.43], *p* < 0.001, adj. OR 0.47, [0.24–0.92], *p* = 0.029) with ICU mortality was confirmed.

**Table 3:** Explanatory variables in simple and multiple logistic regression

Variable	Simple logistic regression		Multiple logistic regression	
	OR [95% CI]	*P* value	OR [95% CI]	*P* value
CFH grouping	3.77 [2.51–5.72]	< 0.001	2.17 [1.11–4.32]	0.025
Hp grouping	0.29 [0.19–0.43]	< 0.001	0.47 [0.24–0.92]	0.029
BMI	1.00 [0.99–1.02]	0.740	0.96 [0.92–1.00]	0.080
pH at ECMO initiation	0.06 [0.01–0.33]	0.002	0.08 [0.01–1.27]	0.080

*CFH* cell-free hemoglobin, *Hp* haptoglobin, *BMI* body mass index, *OR* odds ratio, *CI* confidence interval. Eliminated variables: CCI, inhaled nitric oxide, septic shock, RRT, PRBC units, ARDS etiology, lactate, SOFA, APACHE, SAPS, RASS, pO_2_, PIP, Pplat, PEEP and compliance at ECMO initiation

**Fig. 3:**
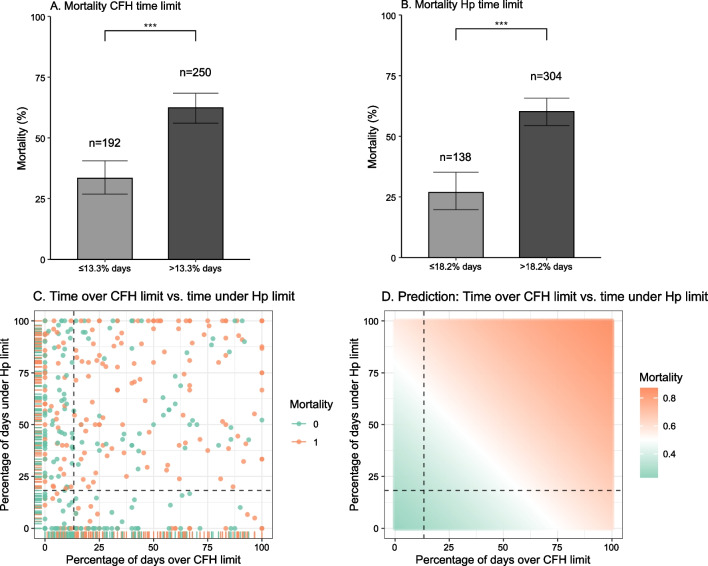
**A** Mortality by CFH time limit. **B** Mortality by Hp time limit. **C** Distribution of patients according to the time spent above the CFH and below the Hp limit, colored by ICU mortality. **D** Prediction of ICU mortality based on the time spent above the CFH and below the Hp limits. Dashed lines represent the calculated time limits for CFH (13.3%) and Hp (18.2%)

Besides the quantitative cutoff values of mean CFH and mean Hp plasma concentrations, which reflect average concentrations over the entire course of V-V ECMO therapy, we hypothesized that a certain time interval spent above a critical CFH plasma concentration or below a potentially protective Hp cutoff value might be associated with an increase in mortality in terms of a cumulative toxic effect. A significant difference in mortality divided the cohort into two groups when the percentage of days over the CFH limit in relation to all therapy days exceeded 13.3% (≤ 13.3% of days with CFH > 11 mg/dl: 33%; [95% CI, 26.81–40.54], *n* = 192 vs. > 13.3% of days with CFH > 11 mg/dl: 62%; [56.05–68.36], *n* = 250, *p* < 0.001) (Fig. 3A). Similarly, a significant increase in mortality was detected at > 18.2% of days below the Hp cutoff value of 0.39 g/l (≤ 18.2% days below the Hp limit: 27%; [95% CI, 19.80–35.14], *n* = 138 vs. > 18.2% days below the Hp limit: 60%; [54.43–65.70], *n* = 304, *p* < 0.001, Fig. 3B). Further splits of the conditional inference trees were not evaluated; however, complete conditional inference trees are provided in the Additional file 1. The correlation between the timespan of CFH above the limit of 11 mg/dl and the timespan of Hp below the limit of 0.39 g/l and ICU mortality is shown in Fig. 3C. A prediction of ICU mortality was attempted with a multiple logistic regression model based on the two time parameters as response variables (Fig. 3D). When a CFH plasma concentration exceeded 50 mg/dl, the cutoff value defined by the Extracorporeal Life Support Organization (ELSO) for moderate hemolysis, the timespan required to reach a significant increase in ICU mortality was reduced to 5.26% of therapy days in our patient cohort (Additional file 1). However, CFH plasma concentrations exceeding 50 mg/dl were only detected in a small fraction of 22 of the included 442 patients.

An attempt to quantify the effects of a single event of hemolysis was made by calculating the cutoff values of maximum CFH and minimum Hp values. The resulting increase in ICU mortality was detected at 25 mg/dl for maximum CFH (max CFH ≤ 25 mg/dl: 37.2%, [95% CI, 31.25–43.55], *n* = 250, *p* < 0.001 vs. max CFH > 25 mg/dl: 66.1%, [58.93–72.71], *n* = 192, *p* < 0.001) and at 0.42 g/l for minimum Hp (mean Hp ≤ 0.42 g/l: 57.6%, [95% CI, 52.13–62.81], *n* = 344 vs. mean Hp > 0.42 g/l: 22.4%, [14.89–32.21], *n* = 98, *p* < 0.001). Corresponding conditional inference trees are provided in the Additional file 1.

## Discussion

In patients with ARDS receiving therapy with V-V ECMO, cutoff values of 11 mg/dl for mean CFH and 0.39 g/l for mean Hp during therapy with V-V ECMO were associated with significant increases in ICU mortality. In addition, critical timespans for CFH concentrations above and Hp concentrations below the respective cutoff values could be determined. Both, CFH and Hp plasma concentrations were independently associated with mortality. In addition, CFH-associated mortality was dependent on Hp plasma concentrations.

The primary objective of this study was to identify cutoff values of mean CFH and mean Hp plasma concentrations that define a significant increase in ICU mortality in patients with ARDS and treatment with V-V ECMO. By calculating cutoff values of a cumulative dose effect of hemolysis, scalable laboratory markers are generated that can be used for prediction of mortality and severity of the disease. Although more heterogeneous than median CFH values, calculation of patients mean CFH was chosen to identify a critical cumulative dose effect of hemolysis, because short but severe elevations of CFH can correlate with and possibly precipitate adverse outcomes.

Previous studies on hemolysis in patients with ARDS and therapy with V-V ECMO revealed significant associations between mortality and CFH plasma concentrations [7, 31, 33]. These studies used mainly singular timepoints for CFH detection and the CFH thresholds used to indicate relevant hemolysis were far above the cutoff for CFH values found in this study [31, 33, 37, 38]. According to the ELSO registry, the complication “moderate hemolysis” in patients treated with ECMO is defined as a CFH value exceeding 50 mg/dl for two consecutive days, “severe hemolysis” as a CFH value exceeding 100 mg/dl for two consecutive days [39]. Consistent with this definition, a mean CFH plasma concentration exceeding 50 mg/dl was associated with a significant increase in mortality in this study cohort. However, using recursive binary partitioning with conditional inference trees, the most significant increase in mortality was already observed at a mean CFH plasma concentration of only 11 mg/dl, suggesting that hemolysis-associated mortality might start at much lower CFH plasma concentrations. The correlation of these lower CFH values—which would be considered even below the threshold for “moderate hemolysis” according to ELSO criteria—with mortality was not only evident for the mean CFH plasma concentration, but also for this threshold being met at only 13% of therapy days for each patient. Additional analysis of maximum CFH indicated that the effects of relevant hemolysis as a one-time event required higher CFH concentrations. Considering CFH as a physiologic contributor to disease severity, the findings of this study imply that hemolysis may have an impact on mortality in patients with ARDS and treatment with V-V ECMO at much lower CFH plasma concentrations than previously thought, and that these low CFH concentrations might contribute to mortality by a cumulative dose effect over time. Therefore, early recognition of significant hemolysis and correction of possible and modifiable causes such as pump head thromboses, the use of unnecessary high blood flows or single lumen cannulas might mitigate the cumulative burden of moderate increased plasma concentrations of CFH [5].

Decreased Hp plasma concentrations are associated with increased mortality in patients with sepsis [2]. In addition, higher plasma levels of Hp seem to protect from hemolysis-associated renal injury in patients with ARDS and treatment with V-V ECMO [4]. Here, we demonstrate that ICU mortality in this patient cohort was inversely and independently associated with Hp plasma concentrations. Based on the physiologic interplay between CFH and Hp, Hp plasma concentrations need to be taken into consideration when the injurious effect of elevated CFH plasma concentrations is evaluated. Furthermore, these data suggest a protective effect of higher Hp plasma concentrations when hemolysis occurs. Supplementation of Hp to counteract increased CFH plasma concentrations and CFH-associated adverse effects including increased mortality, renal injury, or loss of vascular integrity has been successfully tried as a therapeutic intervention in various preclinical models [23, 40–44]. So far, treatment with exogenous Hp is approved in Japan for individual cases of to prevent hemoglobinuria and potentially renal damage in cardiac surgery patients suffering from increased hemolysis after prolonged cardio-pulmonary bypass [45, 46].

### Limitations

Due to the retrospective design of our study, only associations with mortality of a specific patient cohort were detected and no causal conclusions can be drawn. We specifically addressed the effects of hemolysis by focusing on mean CFH and mean Hp plasma concentrations over the timespan of all therapy days of V-V ECMO. However, both parameters can only be calculated retrospectively and might be of limited use in an ex ante approach to trigger potential decisions or interventions. Even though the additional analysis of maximum CFH and minimum Hp concentrations explored the effects of extreme values, patient-specific daily fluctuations were not considered. Only the association of hemolysis with ICU-mortality was studied, but not the many possible causes for hemolysis and their potentially independent association with mortality. Similarly, technical parameters of the ECMO system and changes in ARDS therapy over time were not taken into consideration. In addition, factors that might impact plasma concentration of CFH such as transfusions of PRBCs that have been stored for prolonged intervals or a RRT were eliminated from further analysis during the multivariate logistic regression model with backward variable selection [47]. Therefore, prospective data are needed to explore whether CFH and Hp plasma concentrations play a relevant functional role in the course of ARDS or are primarily markers of disease severity and associated complications.

## Conclusions

Relevant hemolysis that impacts mortality in patients with ARDS and treatment with V-V ECMO may start at relatively low values of CFH that may impact on outcome via a cumulative dose effect. Furthermore, Hp plasma concentrations need to be taken into consideration when injurious effects of elevated CFH plasma concentrations are evaluated. Future clinical trials should address supplementation therapy with exogenous Hp as a therapeutic strategy in patients with severe ARDS and treatment with V-V ECMO.

## Supplementary Information

**Additional file 1.** Potential of cell-free hemoglobin and haptoglobin as prognostic markers in patients with ARDS and treatment with veno-venous ECMO.

## Data Availability

Data are available from the corresponding author on reasonable request.

## Abbreviations

ARDS: Acute respiratory distress syndrome
PRBCs: Packed red blood cells
CFH: Cell-free hemoglobin
mCFH: Mean cell-free hemoglobin
NO: Nitric oxide
Hp: Haptoglobin
mHp: Mean haptoglobin
V-V ECMO: Veno-venous extracorporeal membrane oxygenation
BMI: Body mass index
SOFA: Sequential organ failure assessment
APACHE: Acute physiology and chronic health evaluation
SAPS: Simplified acute physiology score
RASS: Richmond agitation-sedation scale
PIP: Peak inspiratory pressure
Pplat: Plateau pressure
PEEP: Positive end-expiratory pressure
RRT: Renal replacement therapy
ELSO: Extracorporeal Life Support Organization

## References

[1] Larsen R, Gozzelino R, Jeney V (2010). A central role for free heme in the pathogenesis of severe sepsis. Sci Transl Med..

[2] Janz DR, Bastarache JA, Sills G (2013). Association between haptoglobin, hemopexin and mortality in adults with sepsis. Crit Care.

[3] Vermeulen Windsant IC, Hanssen SJ, Buurman WA, Jacobs MJ (2011). Cardiovascular surgery and organ damage: time to reconsider the role of hemolysis. J Thorac Cardiovasc Surg.

[4] Graw JA, Hildebrandt P, Krannich A (2022). The role of cell-free hemoglobin and haptoglobin in acute kidney injury in critically ill adults with ARDS and therapy with VV ECMO. Crit Care.

[5] Materne LA, Hunsicker O, Menk M, Graw JA (2021). Hemolysis in patients with extracorporeal membrane oxygenation therapy for severe acute respiratory distress syndrome—a systematic review of the literature. Int J Med Sci.

[6] Hod EA, Brittenham GM, Billote GB (2011). Transfusion of human volunteers with older, stored red blood cells produces extravascular hemolysis and circulating non–transferrin-bound iron. Blood.

[7] Lehle K, Philipp A, Zeman F (2015). Technical-induced hemolysis in patients with respiratory failure supported with veno-venous ECMO—prevalence and risk factors. PLoS ONE.

[8] Rapido F, Brittenham GM, Bandyopadhyay S (2016). Prolonged red cell storage before transfusion increases extravascular hemolysis. J Clin Invest.

[9] Meyer C, Heiss C, Drexhage C (2010). Hemodialysis-induced release of hemoglobin limits nitric oxide bioavailability and impairs vascular function. J Am Coll Cardiol.

[10] Villagra J, Shiva S, Hunter LA, Machado RF, Gladwin MT, Kato GJ (2007). Platelet activation in patients with sickle disease, hemolysis-associated pulmonary hypertension, and nitric oxide scavenging by cell-free hemoglobin. Blood.

[11] Balla J, Vercellotti GM, Jeney V (2007). Heme, heme oxygenase, and ferritin: how the vascular endothelium survives (and Dies) in an iron-rich environment. Antioxid Redox Signal.

[12] Silliman CC, Moore EE, Kelher MR, Khan SY, Gellar L, Elzi DJ (2011). Identification of lipids that accumulate during the routine storage of prestorage leukoreduced red blood cells and cause acute lung injury: neutral lipids from LR-RBCs cause Ali. Transfusion (Paris).

[13] Gladwin MT, Shizukuda Y, Brown B, Ernst I, Blackwelder WC, Castro O (2004). Pulmonary hypertension as a risk factor for death in patients with sickle cell disease. N Engl J Med.

[14] Baron DM, Yu B, Lei C (2012). Pulmonary hypertension in lambs transfused with stored blood is prevented by breathing nitric oxide. Anesthesiology.

[15] Kehrer JP (2000). The Haber-Weiss reaction and mechanisms of toxicity. Toxicology.

[16] Sadrzadeh SM, Graf E, Panter SS, Hallaway PE, Eaton JW (1984). Hemoglobin. A biologic fenton reagent. J Biol Chem.

[17] Jeney V, Balla J, Yachie A (2002). Pro-oxidant and cytotoxic effects of circulating heme. Blood.

[18] Santoro AM, Lo Giudice MC, D’Urso A, Lauceri R, Purrello R, Milardi D (2012). Cationic porphyrins are reversible proteasome inhibitors. J Am Chem Soc.

[19] Lin T, Kwak YH, Sammy F (2010). Synergistic inflammation is induced by blood degradation products with microbial toll-like receptor agonists and is blocked by hemopexin. J Infect Dis.

[20] Vermeulen Windsant IC, de Wit NCJ, Sertorio JTC (2014). Hemolysis during cardiac surgery is associated with increased intravascular nitric oxide consumption and perioperative kidney and intestinal tissue damage. Front Physiol.

[21] Billings FT, Ball SK, Roberts LJ, Pretorius M (2011). Postoperative acute kidney injury is associated with hemoglobinemia and an enhanced oxidative stress response. Free Radic Biol Med.

[22] Schaer DJ, Buehler PW, Alayash AI, Belcher JD, Vercellotti GM (2013). Hemolysis and free hemoglobin revisited: exploring hemoglobin and hemin scavengers as a novel class of therapeutic proteins. Blood.

[23] Schaer CA, Deuel JW, Schildknecht D (2016). Haptoglobin preserves vascular nitric oxide signaling during hemolysis. Am J Respir Crit Care Med.

[24] Deatrick KB, Galvagno SM, Mazzeffi MA (2020). Pilot study evaluating a non-titrating, weight-based anticoagulation scheme for patients on veno-venous extracorporeal membrane oxygenation. Perfusion.

[25] Bréchot N, Mastroianni C, Schmidt M (2018). Retrieval of severe acute respiratory failure patients on extracorporeal membrane oxygenation: any impact on their outcomes?. J Thorac Cardiovasc Surg.

[26] Cholette JM, Pietropaoli AP, Henrichs KF (2018). Elevated free hemoglobin and decreased haptoglobin levels are associated with adverse clinical outcomes, unfavorable physiologic measures, and altered inflammatory markers in pediatric cardiac surgery patients. Transfusion (Paris).

[27] Marchand A, Galen RS, Van Lente F (1980). The predictive value of serum haptoglobin in hemolytic disease. JAMA.

[28] Nouraie M, Lee JS, Zhang Y (2013). The relationship between the severity of hemolysis, clinical manifestations and risk of death in 415 patients with sickle cell anemia in the US and Europe. Haematologica.

[29] Shaver CM, Upchurch CP, Janz DR (2016). Cell-free hemoglobin: a novel mediator of acute lung injury. Am J Physiol-Lung Cell Mol Physiol.

[30] Janz DR, Ware LB (2015). The role of red blood cells and cell-free hemoglobin in the pathogenesis of ARDS. J Intensive Care.

[31] Omar HR, Mirsaeidi M, Socias S (2015). Plasma free hemoglobin is an independent predictor of mortality among patients on extracorporeal membrane oxygenation support. PLoS ONE.

[32] Pan KC, McKenzie DP, Pellegrino V, Murphy D, Butt W (2016). The meaning of a high plasma free haemoglobin: retrospective review of the prevalence of haemolysis and circuit thrombosis in an adult ECMO centre over 5 years. Perfusion.

[33] Kutleša M, Novokmet A, Mraović RJ, Filar B, Mardešić P, Baršić B (2014). Extracorporeal Membrane oxygenation treatment for H1N1-induced acute respiratory distress syndrome (ARDS): results of the Croatian Referral Center for respiratory ECMO. Int J Artif Organs.

[34] Hunsicker O, Materne L, Bünger V (2020). Lower versus higher hemoglobin threshold for transfusion in ARDS patients with and without ECMO. Crit Care.

[35] Hothorn T, Hornik K, Zeileis A (2006). Unbiased recursive partitioning: a conditional inference framework. J Comput Graph Stat.

[36] Newman DA (2003). Longitudinal modeling with randomly and systematically missing data: a simulation of ad hoc, maximum likelihood, and multiple imputation techniques. Organ Res Methods.

[37] Lorusso R, Gelsomino S, Parise O (2017). Neurologic injury in adults supported with veno-venous extracorporeal membrane oxygenation for respiratory failure: findings from the extracorporeal life support organization database. Crit Care Med.

[38] Mazzeffi M, Kon Z, Menaker J (2019). Large dual-lumen extracorporeal membrane oxygenation cannulas are associated with more intracranial hemorrhage. ASAIO J.

[39] Extracorporeal Life Support Organization. ELSO Registry Data Definitions. Published online May 17, 2022. https://www.elso.org/Portals/0/Files/PDF/ELSO%20Registry%20Data%20Definitions%2005_17_22.pdf.

[40] Remy KE, Cortés-Puch I, Solomon SB (2018). Haptoglobin improves shock, lung injury, and survival in canine pneumonia. JCI Insight..

[41] Schaer CA, Jeger V, Gentinetta T (2021). Haptoglobin treatment prevents cell-free hemoglobin exacerbated mortality in experimental rat sepsis. Intensive Care Med Exp.

[42] Graw JA, Mayeur C, Rosales I (2016). Haptoglobin or hemopexin therapy prevents acute adverse effects of resuscitation after prolonged storage of red cells. Circulation.

[43] Baek JH, D’Agnillo F, Vallelian F (2012). Hemoglobin-driven pathophysiology is an in vivo consequence of the red blood cell storage lesion that can be attenuated in guinea pigs by haptoglobin therapy. J Clin Invest.

[44] Graw JA, Yu B, Rezoagli E (2017). Endothelial dysfunction inhibits the ability of haptoglobin to prevent hemoglobin-induced hypertension. Am J Physiol-Heart Circ Physiol.

[45] Hashimoto K, Nomura K, Nakano M, Sasaki T, Kurosawa H (1993). Pharmacological intervention for renal protection during cardiopulmonary bypass. Heart Vessels.

[46] Tanaka K, Kanamori Y, Sato T (1991). Administration of haptoglobin during cardiopulmonary bypass surgery. ASAIO Trans.

[47] Graw JA, Bünger V, Materne LA (2022). Age of red cells for transfusion and outcomes in patients with ARDS. J Clin Med.

