# Supplementary material for: Potential of cell-free hemoglobin and haptoglobin as prognostic markers in patients with ARDS and treatment with veno-venous ECMO
Source: J Intensive Care. 2023 Apr 20;11:15. doi: 10.1186/s40560-023-00664-5 (PMC10116665; doi:10.1186/s40560-023-00664-5)

Supplementary Information

Potential of cell-free hemoglobin and haptoglobin as prognostic markers in patients with ARDS and treatment with veno-venous ECMO

V. Bünger, O. Hunsicker, A. Krannich, F. Balzer, C.D. Spies, W.M. Kuebler, S. Weber-Carstens, M. Menk, J.A. Graw

Table of Contents

Data Sources 2

Supplemental Figures 3

Supplemental Figure 1: Complete conditional inference trees for ICU mortality. 3

Supplemental Figure 2: Conditional inference trees for ICU mortality. 4

Supplemental Figure 3: ICU mortality and conditional inference tree of the time component for a CFH cut-off value of 50mg/dl.................................. 5

Data Sources

Data on patient demographics, ARDS etiology and comorbidities were extracted from the hospital data management system (SAP, Walldorf, Germany). Further data regarding admission scores, ARDS characteristics, treatment, rescue therapies and medication, ventilation parameters and laboratory parameters were extracted from the electronic ICU data management system used at the hospital (COPRA 5, Sasbachwalden, Germany).

Supplemental Figures

Supplemental Figure 1: Complete conditional inference trees for ICU mortality.

A. Mean CFH as explanatory variable. B. Mean Hp as explanatory variable. C. CFH time component (percentage of days over the CFH limit in relation to all therapy days) as explanatory variable. D. Hp time component (percentage of days under the Hp limit in relation to all therapy days) as explanatory variable.

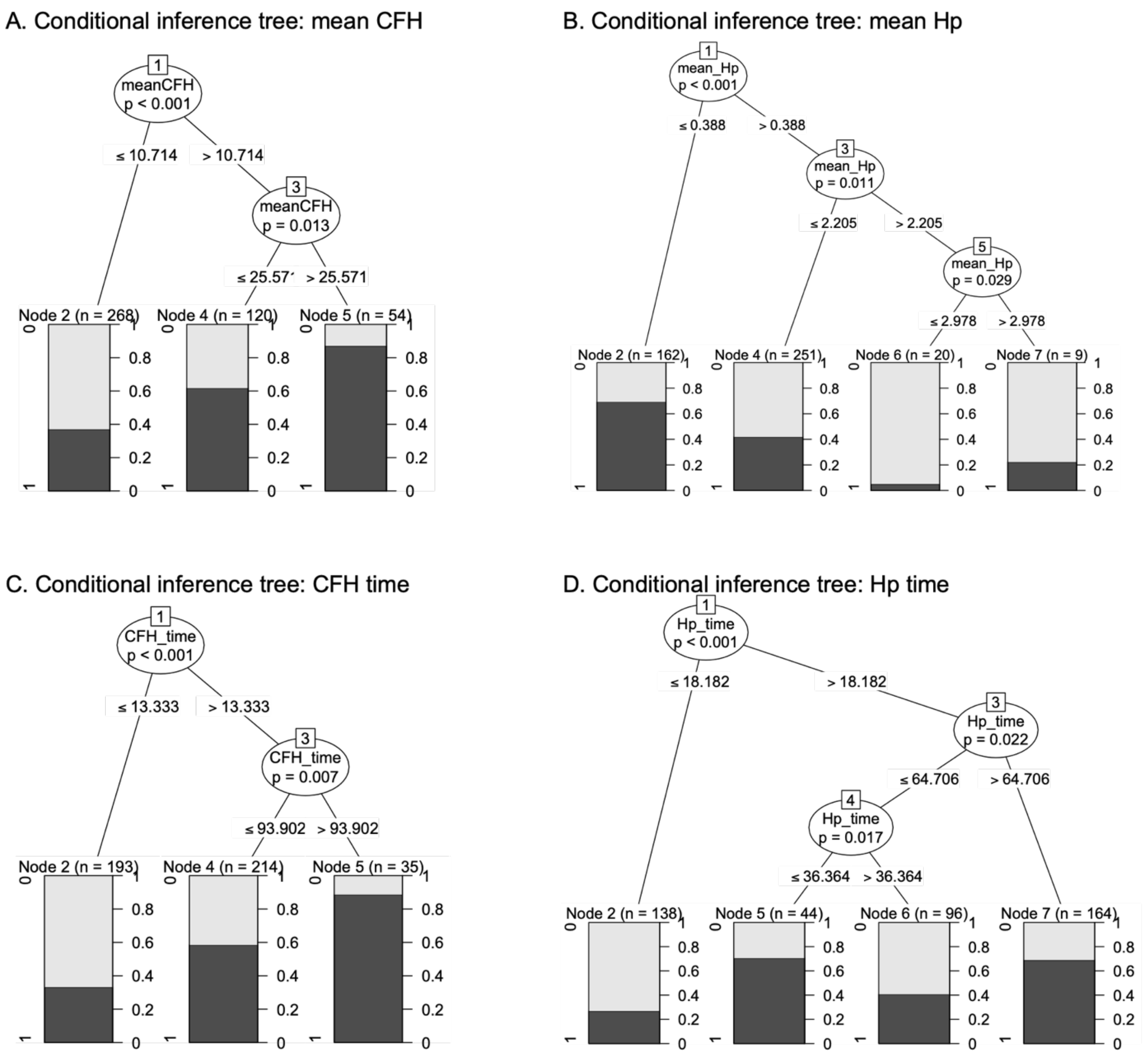

Supplemental Figure 2: Conditional inference trees for ICU mortality.

A. Maximum CFH as explanatory variable. B. Minimum Hp as explanatory variable.

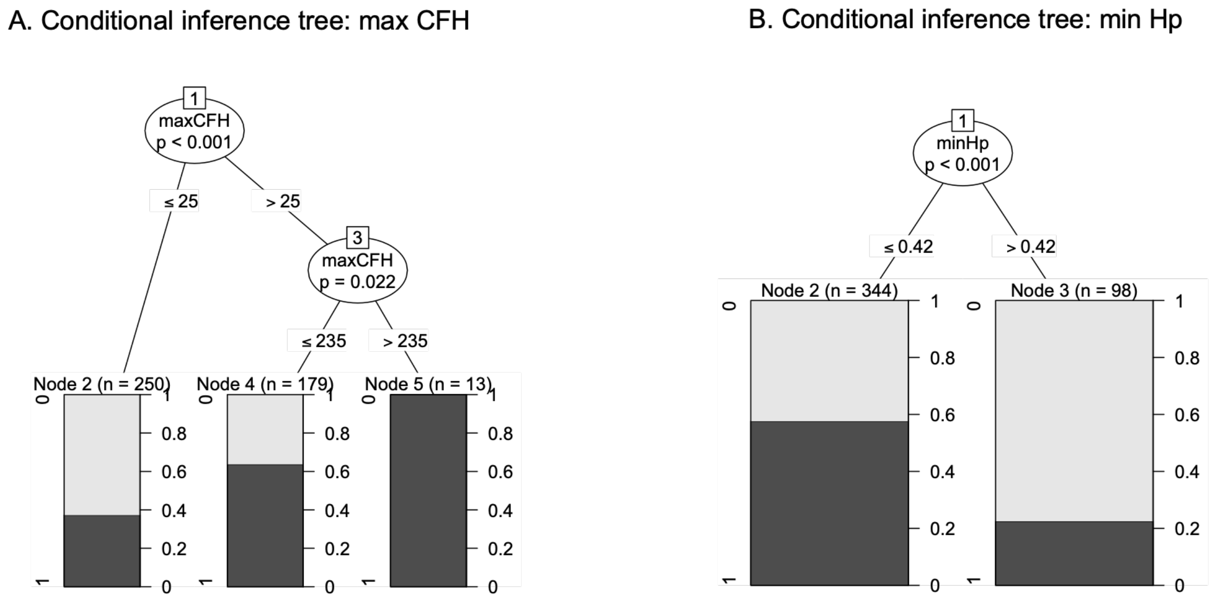

Supplemental Figure 3: ICU mortality and conditional inference tree of the time component for a CFH cut-off value of 50mg/dl.

A. Mortality grouped by mean CFH cut-off value 50 mg/dl. B. Conditional inference tree with ICU mortality as response variable and CFH time component (percentage of days with CFH value over 50 mg/dl in relation to all therapy days).

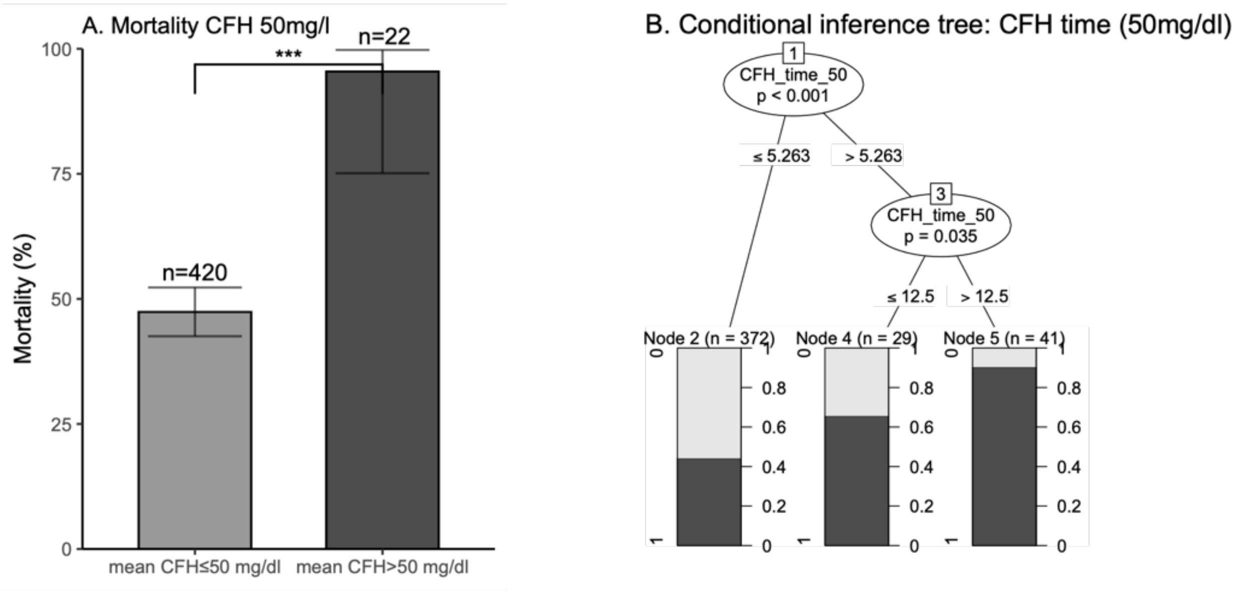

Supplement: Supplementary file 1 — Additional file 1. Potential of cell-free hemoglobin and haptoglobin as prognostic markers in patients with ARDS and treatment with veno-venous ECMO. [file 40560_2023_664_MOESM1_ESM.pdf]
